# The *Odad3* Gene Is Necessary for Spermatozoa Development and Male Fertility in Mice

**DOI:** 10.3390/cells13121053

**Published:** 2024-06-18

**Authors:** Miriam Pasquini, Francesco Chiani, Alessia Gambadoro, Chiara Di Pietro, Renata Paoletti, Tiziana Orsini, Sabrina Putti, Ferdinando Scavizzi, Gina La Sala, Olga Ermakova

**Affiliations:** 1 Institute of Biochemistry and Cell Biology (IBBC), National Research Council of Italy (CNR), Adriano Buzzati-Traverso Campus, Via Ramarini, 32, 00015 Monterotondo, Italy; miriam.pasquini@cnr.it (M.P.); francesco.chiani@cnr.it (F.C.); alessia.gambadoro@cnr.it (A.G.); chiara.dipietro@cnr.it (C.D.P.); renata.paoletii@emma.cnr.it (R.P.); tiziana.orsini@cnr.it (T.O.); sabrina.putti@cnr.it (S.P.); ferdinando.scavizzi@cnr.it (F.S.); 2 European Mouse Mutant Archive (EMMA), INFRAFRONTIER, Monterotondo Mouse Clinic, National Research Council of Italy (CNR), Adriano Buzzati-Traverso Campus, Via Ramarini, 32, 00015 Monterotondo, Italy

**Keywords:** cilia, *Odad3* gene, Primary Ciliary Dyskinesia, mouse model of PCD, male infertility, MMAF, *Odad3* gene dosage

## Abstract

*Odad3* gene loss-of-function mutation leads to Primary Ciliary Dyskinesia (PCD), a disease caused by motile cilia dysfunction. Previously, we demonstrated that knockout of the *Odad3* gene in mice replicates several features of PCD, such as hydrocephalus, defects in left–right body symmetry, and male infertility, with a complete absence of sperm in the reproductive tract. The majority of *Odad3* knockout animals die before sexual maturation due to severe hydrocephalus and failure to thrive, which precludes fertility studies. Here, we performed the expression analysis of the *Odad3* gene during gonad development and in adult testes. We showed that *Odad3* starts its expression during the first wave of spermatogenesis, specifically at the meiotic stage, and that its expression is restricted to the germ cells in the adult testes, suggesting that *Odad3* plays a role in spermatozoa formation. Subsequently, we conditionally deleted the *Odad3* gene in adult males and demonstrated that even partial ablation of the *Odad3* gene leads to asthenoteratozoospermia with multiple morphological abnormalities of sperm flagella (MMAF) in mice. The analysis of the seminiferous tubules in *Odad3*-deficient mice revealed defects in spermatogenesis with accumulation of seminiferous tubules at the spermiogenesis and spermiation phases. Furthermore, analysis of fertility in heterozygous *Odad3^+/−^* knockout mice revealed a reduction in sperm count and motility as well as abnormal sperm morphology. Additionally, *Odad3^+/−^* males exhibited a shorter fertile lifespan. Overall, these results suggest the important role of *Odad3* and *Odad3* gene dosage in male fertility. These findings may have an impact on the genetic and fertility counseling practice of PCD patients carrying *Odad3* loss-of-function mutations.

## 1. Introduction

Motile cilia are present on terminally differentiated Multiciliated Cells (MCCs) in epithelial tissues of the brain ependyma, airway, and reproductive ducts. Additionally, motile cilia also exist as a solitary structure, such as embryonic node cilia and sperm flagella [1]. The directional beating of cilia in various organs and physiological systems creates gradients of biologically active molecules, moves cells and cellular debris along the epithelial surfaces, and expels mucus by moving extracellular fluids. The directional beating of nodal cilia establishes left–right body asymmetry, while flagella beating ensures sperm motility [2].

Defects in normal biological processes that prevent the differentiation of the specialized MCCs or the correct assembly of ciliary axonemes on MCCs lead to a complex genetic disease called Primary Cilia Dyskinesia (PCD; OMIM:244400). To date, mutations in over 40 genes causing PCD have been identified in humans [2,3]. The symptoms of PCD include chronic nasal discharge; ear, nose, and chest infections; pulmonary disease; bronchiectasis. Additionally, half of the patients exhibit situs inversus and, in many cases, infertility has been reported [3].

Both motile cilia and sperm flagella are microtubule-based axoneme. In mammals, the axoneme comprises two central and nine surrounding microtubule doublets, which are connected to the central pair of microtubules by radial spokes [4,5]. Specific components of motile cilia, which are necessary for cilia beatings, include inner dynein arms (IDA) and outer dynein arms (ODA) formed by dynein motor protein complexes. Dynein generates forces by sliding against adjacent peripheral microtubules and controls rhythmic wave movements of cilia in an ATP-dependent manner [6,7]. Multi-subunit dynein arm motor complexes are produced and preassembled in the cytosol, transported to the ciliary or flagella compartments, and anchored into the axonemal microtubule scaffold via the ODA docking complex (ODA–DC) system [6]. Mutations in the proteins, building blocks of axonemal structures, are frequently reported in PCD [2,3].

Male infertility is one of the pathological features described in PCD patients. Motile cilia and sperm flagella have a similar structural organization. In the past, infertility associated with PCD was thought to be due to abnormal sperm motility and morphology, including multiple morphological abnormalities of the sperm flagellum (MMAF) caused by mutations in axonemal genes [3]. Nevertheless, some PCD patients are diagnosed with a complete absence of spermatozoa (azoospermia) or low sperm counts (oligospermia). These findings indicate that PCD infertility cannot be attributed solely to flagellar dysfunction, suggesting the involvement of new paradigms.

Azoospermia is frequently attributed to a blockage in the process of spermatogenesis. Spermatogenesis is a complex developmental program that continuously generates mature spermatozoa in the testes [8]. The process of spermatozoa maturation takes place in the seminiferous tubules, culminating in the release of mature spermatozoa into the lumen of the tubule. In adult mice, the seminiferous tubules undergoing spermatogenesis are divided into 12 stages (IXII) based on the specific arrangements of differentiated germ cells within the seminiferous epithelium of the testicular tubules. A single tubule section may contain four to five generations of germ cells at any given time [9,10,11]. Stages I–VIII are characterized by the presence of two generations of spermatids, namely round spermatids (RSs) and elongated spermatids (ESs), within a cross-section of a single seminiferous tubule, while only elongated spermatids are present in the tubule at stages IX–XII [10]. Stages I–VIII of spermatogenesis are defined as spermiogenesis [12]. During the process of spermiogenesis, the RSs undergo significant cytoplasmic changes and are transformed into the ESs. The centrally located nucleus of the RS undergoes a series of transformations, it moves to the periphery, becomes condensed, elongates, and ultimately shapes into the head of the spermatozoon. Additionally, the central portion and the secondary structures of the sperm flagellum begin to assemble [13]. The final remodeling of the mature elongated spermatid occurs during the spermiation stage VII–VIII. At this stage, the spermatids align along the luminal edge and are ultimately released into the tubule lumen before passing to the epididymis [9,12]. Initially, the spermatozoa released from the tubular epithelium are immotile. Subsequently, the spermatozoa are collected in the rete testis and transported to the epididymis via efferent ducts (EDs) [14,15]. As they pass through the epididymis, the spermatozoa gradually mature and gain full motility. The mature sperms are stored in the cauda before being released [16].

In the male reproductive tract, motile cilia are only detected on the epithelial surface of efferent ducts [15]. Previously, it was assumed that the primary function of these cilia was to facilitate the directional transport of immotile sperm from the rete testes to the epididymis. However, recent studies demonstrated that these cilia do not beat uniformly in one direction but instead generate turbulence by beating in a whip-like motion with continuous changes in the direction. This generates a centripetal force that maintains spermatozoa in suspension within the efferent duct lumen [17]. The loss of efferent duct motile cilia or their abnormal beating causes sperm aggregation and agglutination, luminal obstruction, and the formation of sperm granulomas, which, in turn, induce back-pressure atrophy of the testes and ultimately azoospermia [17,18,19].

ODAD3, previously known as CCDC151, is a component of the pentameric Outer Dynein Arm Docking Complex (ODA–DC) of the mammalian axonemes of both motile cilia and sperm flagella [20,21,22]. The loss-of-function mutations in *Odad3* gene result in ciliary dysfunctions and cause PCD in humans and mice [21,23,24,25,26]. The main pathological features of PCD, such as bronchiectasis, sinusitis, and situs inversus, were observed in patients carrying mutations in the *Odad3* gene. However, infertility problems have not been reported thus far, likely due to the young age of patients, who have not yet reached sexual maturity.

In addition to ODAD3, the complex is also composed of ODAD1 (CCDC114); ODAD2 (ARMC4) and ODAD4 (TTC25) subunits. The subunits of the docking complex have been identified in both mammalian respiratory cilia and sperm flagella [20,27]. Homozygous mutation in *ODAD1* (*CCDC114*) is associated with PCD, which affects the airways in approximately 30% of patients presenting situs inversus [28,29]. The infertility issue is reported in only 11% of female patients, but not in males. However, the majority of patients in the study group did not reach the age of sexual maturation [28]. Mutations in the ODAD2 (ARMC4) component of the ODA–DC complex have also been described in patients with PCD, but the impact of the mutation on fertility has not been described [30,31,32,33]. Importantly, genetic ablation of the *Drosophila Gudu* gene, which is highly homologous to vertebrate *ARMC4*, affects fertility due to a block in spermatogenesis and the failure to produce motile individual spermatozoa [34]. Another component of the ODA–DC complex ODAD4, (TTC25) has also been identified as mutated in patients with PCD [35,36,37]. In humans, low expression of *TTC25* was detected during the whole process of sperm development, suggesting a potentially significant role for the *TTC25* gene in spermatogenesis [38].

Here, we studied the role of the *Odad3* gene in male infertility using *Odad3* mutant mouse models. Previously, we demonstrated that *Odad3* homozygous knockout mice die before reaching sexual maturity, exhibiting severe hydrocephalus and growth delay [24]. In three males that survived to sexual maturity, analysis of the epididymal ducts and cauda revealed the complete absence of spermatozoa, demonstrating that *Odad3* gene knockout causes azoospermia in mice.

In this study, we used a conditional knockout strategy to analyze the role of the *Odad3* gene in spermatozoa development and maturation. Our data showed that even partial ablation of the *Odad3* gene in adult testes results in low motile sperm counts, abnormal sperm motility and morphology, conditions defined as oligoasthenoteratozoospermia (OATS). Our results suggest that this condition might be caused by impaired spermatogenesis at the spermiogenesis and spermiation phases in adult murine mutant testes. Importantly, heterozygous *Odad3* knockout males also have OATS and show a premature decline in fertility compared to their wild-type littermates. Overall, our results demonstrate that the gene dosage of *Odad3* controls male fertility in mice. 

## 2. Materials and Methods

### 2.1. Ethics Statement

All animals in this study were handled in accordance with the experimental protocols and animal care procedures, reviewed and approved by the Ethical and Scientific Commission of the Veterinary Health and Welfare Department of the Italian Ministry of Health (protocol approval reference: protocol approval reference: N:0000688 03/21/2008. The ethical and safety rules and guidelines for the use of animals in biomedical research are provided by the Italian laws and regulations, in the application of the relevant European Union’s directives (n. 86/609/EEC and 2010/63/EU). 

### 2.2. Animals

All animals in this study were produced and maintained on a C57BL/6N background by the European Mouse Mutant Archive (EMMA) facility in Monterotondo, Roma, Italy. Animals were maintained in a temperature-controlled room at 21 ± 2 °C, on a 12 h light–dark cycle (lights on at 7 a.m. and off at 7 p.m.). After weaning, mice were housed by the litter of the same sex, 3 to 5 per cage, and provided with food and water available ad libitum in a specific pathogen-free facility. The *Odad3^tm1b/+^* mice (formerly called *Ccdc151 ^tm1b/+^* were produced by the IKMC consortium at Monterotondo, Italy described by Chiani et al. [24] and in Supplementary Figure S1. Genotyping of the *Odad3^tm1b^* allele was performed with the following primers: *Odad3-F*: AGAGCCCTGGATCTTAACTGCTGA; *Odad3-*R: TCCAAGTCATGCAGAGCTGGGATT and *Frt-Rev*: CCTTCCTCCTACATAGTTGGCAGT. The wild-type allele produces a PCR product of 307 bp, while the *Odad3 ^tm1b^* allele is 270 bp in size. The generation of the animals with the conditional knockout allele was described by Chiani et al. [24] and in Supplementary Figure S2. The animals homozygous (experimental group) or heterozygous (control group) for the *Odad3* conditional allele were also heterozygous for the *ROSA26ERT2-Cre* allele (MGI:3764519) for the induction of the Cre recombinase by tamoxifen. The genotyping of the conditional allele was carried out as follows: *Odad3-F*: AGAGCCCTGGATCTTAACTGCTGA; *Odad3-R*: TCCAAGTCATGCAGAGCTGGGATT. The size of the bands for the wild-type allele is 305 bp while the conditional knockout allele is 481 bp.

### 2.3. Tissue RNA Extraction and q-PCR Analysis

RNA was extracted from mouse tissues using Trizol Protocol (TRIzol™ Reagent, Invitrogen, ThermoFisher Scientific, Catalog number: 15596026, Waltham, MA, USA) according to the manufacturer’s recommendations. The RNA was treated with DNAse (Zymo Research, DNAse I set, E1010, Irvine, CA, USA) for 1 h at 37 °C following inactivation by adjusting EDTA to 25 mM and incubation at 65 °C for 10 min. The 1 μg of RNA was reverse transcribed using recombinant M-MuLV Reverse Transcriptase (ThermoFisher Scientific, First-strand cDNA Synthesis kit, K1612, Waltham, MA, USA) and random hexamer primers according to the manufacturer’s recommendation. q-PCR was performed using KapaBioSystem SYBR-green reagent (Sigma-Aldrich, KK4601, St. Louis, MO, USA) using an Applied Biosystem real-time system, following standard protocols. We used the following primers for the *Odad3* gene: *Odad3-F:* TCTTGGACCTCTGCCATCTC; *Odad3-R:* GGAGCTCCCAATGGAACAT. The murine *Hprt1* gene was used as a housekeeping gene for relative q-PCR analysis: *Hprt*-*F*: CCTGGTTAAGCAGTACAGCC; *Hprt*-*R*: GAT GGCCACAGGACTAGAAC.

### 2.4. Whole-Mount β-Galactosidase Gene Reporter Assay with X-gal (X-gal/FCN Protocol)

The animals were perfused as described above; the testes were then dissected and fixed in 4% PFA on ice for 30 min. We then assayed the β-galactosidase activity as described previously [39].

### 2.5. Induction of ROSA26ERT2-Cre Recombinase Expression by Tamoxifen in Animals with Odad3 Conditional Knockout Allele

Tamoxifen (Sigma-Aldrich, T5648, St. Louis, MO, USA) dissolved in corn oil (Sigma-Aldrich, C8267, St. Louis, MO, USA) at 10 mg/mL was administered to 4-month-old males at 1 mg per day by intraperitoneal injection for 5 consecutive days (Supplementary Figure S2). Analysis of spermatogenesis was performed at 4 months after tamoxifen injections.

### 2.6. Histological and Immunofluorescence Analysis (IF)

The testes were dissected, washed with PBS, and immediately fixed in 4% wt/vol paraformaldehyde, dehydrated, and embedded in paraffin. Serial sections were prepared by microtome sectioning at 8 µm thickness. Paraffin-embedded testes sections were deparaffinized in exchange wash of xylene two times, then they were gradually rehydrated using decreasing ethanol concentrations followed by PBS (pH = 7.4). H&E staining and IF analysis of testicular sections were performed. For IF analysis, antigen retrieval was performed by boiling the tissue sections in Tris-EDTA buffer at PH 9 (with 0.05% TWEEN 20). Then, the tissue sections were permeabilized with 0.2% Triton X-100 for 10 min, placed in humidity chambers and incubated in 1× PBS, 3% BSA, 5% normal goat serum, 0.05% TWEEN-20 for 1 h at room temperature. Single or double immunofluorescence was performed by incubating the cross-section overnight at 4 °C with the following primary antibodies: mouse y-H2AX (2 μg/mL, Abcam, ab26350, Cambridge, UK) dilution 1:500 or rabbit SOX9 (2 μg/mL, C-20, Santa Cruz Biotechnology, SC-17341, Santa Cruz, CA, USA) dilution 1:100 or with anti-acetylated tubulin (Sigma-Aldrich, T7451, St. Louis, MO, USA) dilution 1:500. All antibody dilutions were performed in PBS with 3% BSA and 0.05% TWEEN 20. The secondary antibodies we used were Alexa Fluor 488-conjugated (Alexa Fluor 488-conjugated Goat anti-Mouse IgG, 1:800, ThermoFisher Scientific, A-11029, Waltham, MA, USA) or rabbit Alexa Fluor 555-conjugated antibodies (Alexa Fluor 555-conjugated Donkey anti-Rabbit IgG, dilution 1:800, ThermoFisher Scientific, A-31572, Waltham, MA, USA), used to visualize y-H2AX and SOX9, respectively, and Alexa Fluor 555 (Alexa Fluor 555-conjugated Donkey anti-Mouse IgG, dilution 1:800, ThermoFisher Scientific, A-31572, Waltham, MA, USA) used to visualize anti-acetylated-α-tubulin antibodies. The nuclei were counterstained with 5 μM DAPI (ThermoFisher Scientific, Waltham, MA, USA) dissolved in PBS. Finally, slides were mounted with ProLong Gold (Invitrogen, ThermoFisher Scientific, P36934, Waltham, MA, USA). 

### 2.7. Analysis of Seminiferous Tubule Morphology

Quantification of tubule diameter, lumen diameter, and epithelium thickness was performed as described by Hoque et al. [19]. Briefly, the testes were cross-sectioned and stained with hematoxylin and eosin (H&E). Bright-field images of the whole-testes cross-sections were acquired using a motorized scope (LMD 7000, Leica Microsystem, Wetzlar, Germany) equipped with a 20× dry objective (HC PL FLUOTAR 20× NA: 0.4, Leica Microsystems, Wetzlar, Germany) objective. Images were imported into Image J version 1.53k (National Institute of Health, Bethesda, MA, USA). Outlines were drawn manually around the seminiferous tubule and the lumen. The following equations were used for quantification: tubule diameter = circumference of tubule/π; lumen diameter = circumference of lumen/π; epithelial thickness = tubule diameter-lumen diameter/2; at least 30 transverse sections of seminiferous tubules that were round or nearly round were chosen randomly and measured for each animal. 

### 2.8. Analysis of Sperm Counts and Motility

For each male, a cauda epididymis was transferred to a sperm collection dish containing 1 mL of PBS + BSA1%. After making some cuts in tissue, using a new 16-gauge needle, spermatozoa were dispersed for 10 min at 37 °C.

The tissue was then removed and sperm concentration, expressed as the number of spermatozoa per mL, was determined by counting the cells in 10 or more squares of a Mekler Counting Chamber (Sefi-Medical Instruments, Ltd., Haifa, Israel) three times. The mean value was multiplied by 100.000 to obtain the concentration in millions per milliliter. Sperm motility was evaluated during sperm count. Progressive and non-progressive (vibrating) spermatozoa were calculated as percentages. Progressive motility refers to sperm that are swimming in a mostly straight line or in very large circles. Non-progressive motility refers to sperm that move but do not make forward progression or swim in very tight circles as described by Elia et al. [40]. Total motility was calculated as the percentage of sperm making any sort of movement, progressive and non-progressive.

### 2.9. Sperm Morphological Analysis 

Morphological evaluation of spermatozoa was conducted using smear preparations. Sperm samples were collected in 1 mL of medium and incubated at 37 °C for 1 h to allow for equilibration. Subsequently, the samples were diluted in a 1:5 ratio with 1× PBS and then gently smeared onto microscope slides which were air-dried at room temperature. The smears were fixed using a solution of 5% acetic acid in 95% ethanol for a period of 3 min. Following fixation, the slides were briefly rinsed in 70% ethanol and air-dried. For staining, spermatozoa were treated with 1% eosin for 10 min, rinsed with 70% ethanol and then air-dried. Morphological analysis was performed utilizing bright-field microscopy. A minimum of 200 sperm cells per specimen were examined and categorized, and the following criteria were applied: normal, folded tails (folded), flagella without head (headless), abnormal heads, sperms with two heads (amorphous) and sperm without tail (head).

### 2.10. Image Acquisition

Fluorescence micrographs were acquired with a motorized LMD7000 microscope (Leica Microsystems) using the manufacturer’s imaging software Leica application Suite X 3.6.0.20104. Confocal images were acquired using an Olympus confocal microscope (Olympus FV1200, Olympus, Tokyo, Japan) equipped with 40× oil immersion objective (UPL FLN 40XO NA:1.30, Olympus, Tokyo, Japan) and visualized with FV10-ASW software (version 4.2; Olympus, Tokyo, Japan). Confocal through-focus image sequences (z-stacks) were collected with steps of 1 µm along the optical axis for a total of 18 µm of optical depth.

## 3. Results

### 3.1. Analysis of Odad3 Expression in Murine Testes

We have previously demonstrated, using mice with the reporter *Odad3-LacZ* gene in which *Odad3* expression was revealed by X-gal staining of whole-mount testes, that *Odad3* is strongly expressed in the adult testes [24]. Here, our investigation focuses on the developmental stages and cellular compartments where *Odad3* exerts its function.

**Figure 1:**
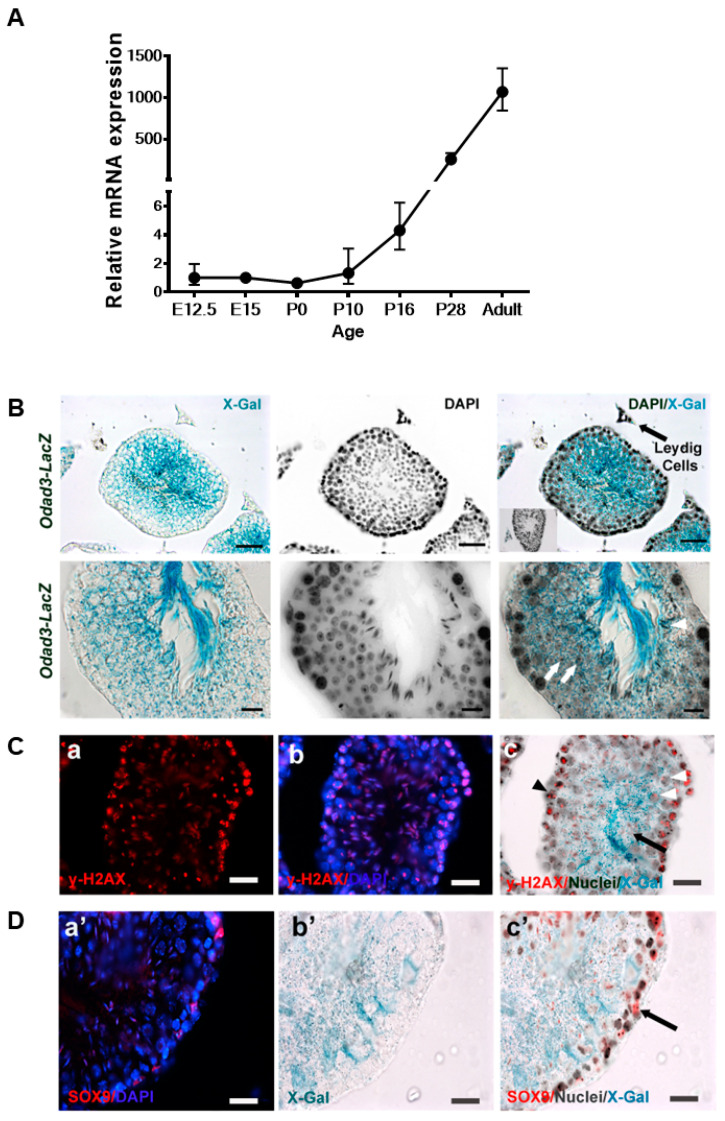
Expression analysis of *Odad3* during development and in adult testes. (**A**) Quantitative PCR analysis of the *Odad3* transcript during development. (**B**) Histological analysis of *Odad3* expression in adult murine testes. Cellular *Odad3* expression was visualized by the analysis of β-galactosidase activity using X-gal staining in *Odad3-LacZ* and wild-type (WT) testes. Left panels—X-gal staining; middle panels—DAPI nuclear staining; right panels—DAPI/X-gal overlay. Upper row image scale bar: 25 μm; lower row scale bar: 10 μm. Arrows indicate round spermatids; arrowhead indicates elongated spermatids Insert in the upper right image is a seminiferous tubule of WT testes—DAPI/X-gal overlay (insert’s magnification 40×). No X-gal activity was detected in the WT testicular epithelium. (**C**) IF analysis of testicular section with y-H2AX antibodies: (**a**) y-H2AX staining (red); (**b**) cellular nuclei were counterstained with DAPI (blue); (**c**) y-H2AX/Nuclei/X-gal overlay, y-H2AX staining is red, DAPI nuclear staining is black for presentation purposes and X-gal is blue. The white arrowheads indicate X-gal and y-H2AX double-positive primary spermatocytes at the pachytene stage of meiosis I. The black arrowhead points to double-positive primary spermatocytes at the leptotene stage of meiosis I. The black arrow indicates elongated spermatid. Elongated spermatids are weakly positive for y-H2AX and X-gal staining. Scale bar: 12.5 μm. (**D**) IF staining of Sertoli cells with SOX9: (**a’**) Sox9 staining (red), nuclear DAPI staining (blue); (**b’**), X-gal staining (blue); (**c’**), SOX9/Nuclei/X-gal overlay: Sox9 immunostaining is red; DAPI-stained nuclei are in black and X-gal staining is blue. The double positive for Sox9 and X-gal Sertoli cells were not detected. The arrow indicates Sertoli Sox9 immunopositive and X-gal negative cell. Scale bar: 12.5 μm.

We first analyzed the expression level of *Odad3* in developing gonads (gametogenesis) using quantitative RT-PCR (q-PCR) analysis. Embryo-fetal gonad development was examined at embryonic day E12.5 and E15 and at birth (postnatal day PND 0). The *Odad3* transcript is not detected either during embryonic development or at birth (Figure 1A), indicating that the *Odad3* gene is not required for the specification of testicular germ cells in the embryo-fetal stage.

In mice, the first wave of spermatogenesis begins at postnatal day (PND) 2–3, when primitive spermatogonia (Type A) synchronously differentiate and new populations of germ cells appear sequentially as follows: spermatocytes at PND 10–14, spermatids at PND 20–28 and testicular spermatozoa at PND 30–35 [41]. The q-PCR analysis revealed that *Odad3* mRNA is first detectable in the testes at PND 10, which coincides with the appearance of spermatocytes. The expression of *Odad3* increases significantly by PND 16 and reaches its maximal expression level in the mature testes (Figure 1A). The results indicate that *Odad3* expression is regulated during the first wave of spermatogenesis and might be critical for spermatocytes differentiation and/or maturation. 

To elucidate the cellular localization of the *Odad3* gene (germinal or somatic) within the adult testes (8 weeks old), a genetic reporter approach was employed, utilizing mice carrying the *Odad3-LacZ* reporter allele. This strategy facilitated the identification of the cells expressing *Odad3*, through the histochemical detection of β-galactosidase activity produced from the *LacZ* gene driven by the *Odad3* promoter (Supplementary Figure S1 and described by Chiani et al.) [24]. Histochemical staining with X-gal on testicular cross-sections from heterozygous *Odad3-LacZ* mice revealed pronounced β-galactosidase activity within the seminiferous tubules (Figure 1B). *Odad3* expression was detected in germ cells located towards the lumen, where the cells at more advanced stages of spermatogenesis are located. Conversely, the germ cells residing near the basal membrane did not exhibit such activity, suggesting an absence of *Odad3* gene expression in germ cells at earlier stages. This spatial expression pattern suggests that *Odad3* gene activation begins during the terminal spermatogonial stages of spermatogenesis and continues through the subsequent stages of spermatogenesis, culminating in the formation of mature spermatozoa. 

To dissect the specific stage of spermatogenic progression at which *Odad3* is localized, we performed X-gal staining coupled with immunofluorescence (IF) analysis using y-H2AX, a marker highly specific to meiotic spermatocytes [42]. Pronounced y-H2AX immunoreactivity congruent with β-galactosidase activity, was detected at the pachytene stage, and persisted until the end of the meiotic process (Figure 1C). Immunofluorescence staining, with SOX9, a specific marker for Sertoli Cells (SC) did not reveal β-galactosidase activity within the Sertoli cell population (Figure 1D). The Leydig cells, which are responsible for testosterone synthesis and are interspersed between seminiferous tubules as isolated cells, exhibited a faint signal in both *Odad3-LacZ* and wild-type testes. Therefore, we concluded that the β-galactosidase signal observed in these Leydig cells was unspecific (Figure 1B). The similar unspecific signal in Leydig cells in the absence of LacZ transgene was also observed by different group [43]. Overall, our results demonstrate the specificity of *Odad3* expression in the germinal cells in testes. 

### 3.2. Conditional Ablation of the Odad3 Gene in Adult Males Results in Reduced Motility and Morphological Abnormalities of Spermatozoa

**Figure 2:**
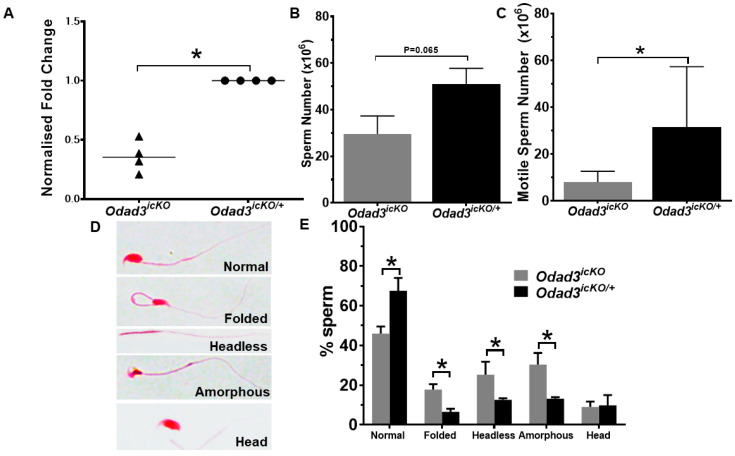
Analysis of spermatozoa from caudal epididymis of animals with the conditional *Odad3* deletion. (**A**) Quantitative PCR analysis of the *Odad3* transcript from homozygous *Odad3^icKO^* and heterozygous *Odad3^icKO/+^* testes. All animals are also heterozygous for the *ROSA26ERT2-Cre* allele, for induction of the conditional deletion by tamoxifen. Error bars represent the mean ± SEM; N = 4 per genotype; unpaired two-tailed *t*-test * *p* = 0.028. (**B**) Analysis of sperm counts prepared from caudal epididymis of *Odad3^icKO^* and *Odad3^icKO/+^* males. Error bars represent the mean ± SEM; unpaired two-tailed *t*-test analysis was performed; N = 7 animals per independent group/genotype were analyzed. (**C**) Motile sperm counts. Error bars represent the mean ± SEM; N = 7 per independent group; unpaired two-tailed *t*-test * *p* = 0.039. (**D**) H&E staining of spermatozoa spread. The representative images of different morphological groups of analyzed spermatozoa are presented. The magnification of the images is 40×. (**E**) Quantification of morphological analysis of the spermatozoa spreads isolated from cauda. At least 150 sperm per independent sample (animal) were counted. Error bars represent the mean ± SEM; N = 4 per genotype; unpaired two-tailed *t*-test: normal sperm—* *p* = 0.026; folded—* *p* = 0.012; headless—* *p* = 0.016; amorphous—* *p* = 0.044.

To elucidate the mechanisms underlying infertility caused by *Odad* loss of function, we employed a conditional gene knockout approach. *Odad3* was deleted in the adult males using Cre-mediated deletion of the *LoxP* floxed exons 2 and 3 of the *Odad3* gene in the animals also carrying *ROSA26ERT2-Cre* allele (MGI:3764519) (Supplementary Figure S2 and described by Chiani et al.) [24]. Cre expression was induced by tamoxifen injection in sexually mature animals. We first analyze the efficiency of Cre-mediated *Odad3* gene deletion upon tamoxifen injection by q-PCR analysis. To do this, we measured *Odad3* transcript in RNA pool extracted from testis of homozygous for the conditional knockout allele animals (*Odad3^icKO^*) and compare it to the heterozygous (*Odad3^icKO/+^*) control animals. The q-PCR analysis revealed a significant reduction in *Odad3* transcript in the *Odad3^icKO^* animals (Figure 2A). However, the analysis also showed an incomplete deletion of the *Odad3* transcript in the mutant animals, with an expression ranging from 20% to 50% of *Odad3* transcript in *Odad3^icKO^* testes compared to *Odad3^icKO/+^*.

We then asked whether the differences in the level of *Odad3* transcript translates into phenotypic differences between *Odad3^icKO^* (low level of the *Odad3* transcript) and *Odad3^icKO/+^* (higher level of the *Odad3* transcript) experimental animal groups. To answer this question, we first analyzed the sperm count, motility and morphology. We observed that conditional homozygous deletion of the *Odad3* gene in *Odad3^icKO^*animals resulted in a tendency for a decrease in the sperm counts (*p* value 0.065) and a statistically significant reduction in the number of motile sperm isolated from the cauda when compared to heterozygous *Odad3^icKO/+^* conditional knockout animals (Figure 2A,B). Subsequently, we conducted a morphological analysis of the sperm. Sperm spreads, from cauda, stained with hematoxylin and eosin (H&E), were examined under brightfield microscopy. The spermatozoa were separated into several morphological classes: normal, folded tails (folded), flagella without a head (headless), abnormal heads, sperm with two heads and amorphous (amorphous) and sperm without a tail (head) (Figure 2C). Quantitative analysis revealed a significant decrease in the percentage of morphologically normal spermatozoa in *Odad3^icKO^* animals with a concomitant increase in the percentage of the spermatozoa with folded flagella, headless and spermatozoa with an amorphous shape, compared to *Odad3^icKO/+^* animals (Figure 2D). These findings suggest that *Odad3* plays a critical role in maintaining normal sperm motility, and morphology and its even incomplete deletion leads to sperm abnormalities, which can be classified as oligoasthenoteratozoospermia with multiple morphological abnormalities of sperm flagella (MMAF).

### 3.3. Aberrant Morphology of Seminiferous Tubules in the Testes of Odad3^icKO^ Animals 

**Figure 3:**
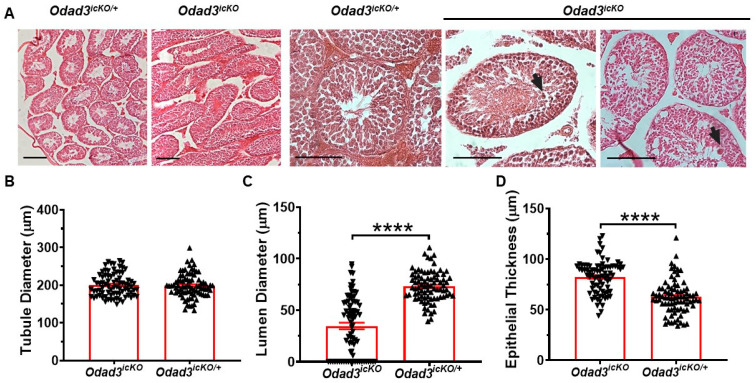
Histological analysis of seminiferous tubules in *Odad3^icKO^* animals. (**A**) H&E staining of the testicular sections from *Odad3^icKO^* and *Odad3^icKO/+^* demonstrated the clogged seminiferous tubules in *Odad3^icKO^* males. Scale bar: 50 μm. Arrows indicate sloughed round spermatids. (**B**–**D**) Quantitative analysis of the morphology of the seminiferous tubules: tubule diameter (**B**), luminal diameter (**C**), and epithelial thickness (**D**). Error bars represent the mean ± SEM; N = 4 per genotype; unpaired two-tailed *t*-test **** *p* ≤ 0.0001.

To understand the defects leading to OATS following the ablation of *Odad3*, we performed histological analysis of the testicular cross-sections prepared from *Odad3^icKO^* and *Odad3^icKO/+^* males (Figure 3A). The histological analysis revealed that numerous seminiferous tubules in the testes of *Odad3^icKO^* males lacked a defined luminal space. Instead, the luminal space is filled with the germ cells sloughing into the center of the tubular space. In addition, we observed an excessive vacuolization of the seminiferous tubule epithelium. To quantify this observation, we measured several parameters: the diameter of seminiferous tubules, the luminal diameter, and the epithelial thickness (Figure 3B–D). Our measurement demonstrated that while the overall diameter was not changed between experimental *Odad3^icKO^* and control *Odad3^icKO/+^* animals (Figure 3B), there was a significant reduction in the luminal diameter of seminiferous tubules in experimental group (Figure 3C) and a corresponding increase in the epithelial thickness compared to control group (Figure 3D).

Overall, these findings suggest the important role of *Odad3* gene dosage for the preservation of structural integrity of the seminiferous tubule in testes.

### 3.4. Reduced Levels of the Odad3 Gene in the Testes Lead to Defects in Spermatogenesis at Spermiogenesis Stages

**Figure 4:**
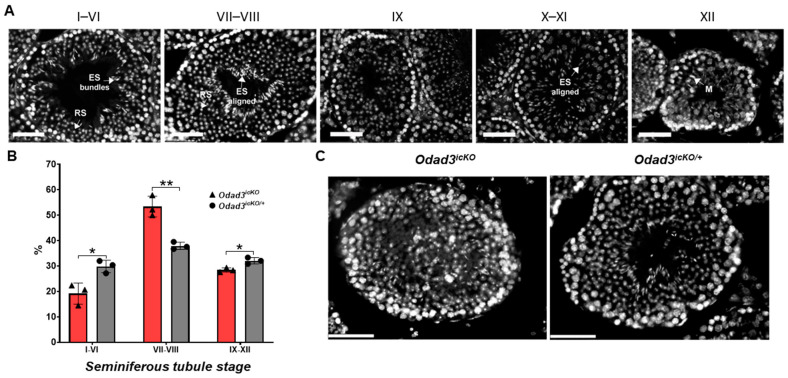
Histological staging analysis of seminiferous tubule cross-sections. (**A**) Stage analysis of the DAPI-stained seminiferous tubule cross-sections from the wild-type animals. Examples of seminiferous tubules at different stages. Stages I–VIII contain two generations of spermatids: round spermatids (RS) and elongated spermatids (ES). In stages I–VI, the elongated spermatids do not line the lumen yet and appear in bundles. During stages VII–VIII, elongated spermatids lined the lumen. In seminiferous tubules staged as IX–XII, no RSs are present. Stage IX is characterized by the start of spermatid elongation and no visible ES; Stages X–XI are characterized by elongates spermatids that are not fully condensed and have a hooked tip. Stages XII—presence of cells undergoing meiotic divisions (M). Scale bar: 50 μm. (**B**) Quantitative analysis of seminiferous tubule staging. Stages IX–XII were counted together since we initially divided between tubules with two types of spermatids and stages where only one type of spermatids is detected. At least 100 seminiferous tubules from *Odad3^icKO^* (N = 3) and *Odad3^icKO/+^* (N = 3) animals were counted. Error bars represent the mean ± SEM; unpaired two-tailed *t*-test, stages I–VI * *p* = 0.0187; stages VII–VIII ** *p* = 0.003; stages IX–XII * *p* = 0.014. (**C**) Representative image of DAPI-stained seminiferous tubule at VII–VIII stage in *Odad3^icKO^* and *Odad3^icKO/+^* testes. Scale bar: 50 μm.

To determine the effect of the *Odad3* gene ablation on the progression of spermatogenesis we quantified the distribution of seminiferous tubules at the different stages of the germ cell development. The stages were delineated as I–VI, where two generations of spermatids (round and elongated) do not line the tubular lumen yet, stages VII–VIII when elongated spermatids polarized and lined the epithelial lumen (spermiation), and stages IX–XII with no round spermatids observed [10]. The staging was performed using DAPI staining of the testicular cross-sections as described by Nakata et al. [44] (Figure 4A). Our analyses revealed an increase in the percentage of the seminiferous tubules at stages VII–VIII and a decrease in the percentage of seminiferous tubules at stages I–VI and IX–XII in *Odad3^icKO^* animals (Figure 4B). Moreover, a subset of the seminiferous tubules at stages VII–VIII exhibited a lumen filled with the round spermatids (Figure 4C). 

These findings suggest a stage-specific defect in normal progression of the testicular germ cell differentiation, highlighting the necessity of the *Odad3* gene dosage for the completion of the spermiation process and the differentiation of round spermatids (RS) into elongated spermatids (ES), and thus for the maturation of spermatozoa.

### 3.5. Deletion of the Odad3 Gene Does Not Grossly Affect Ciliogenesis in ED

**Figure 5:**
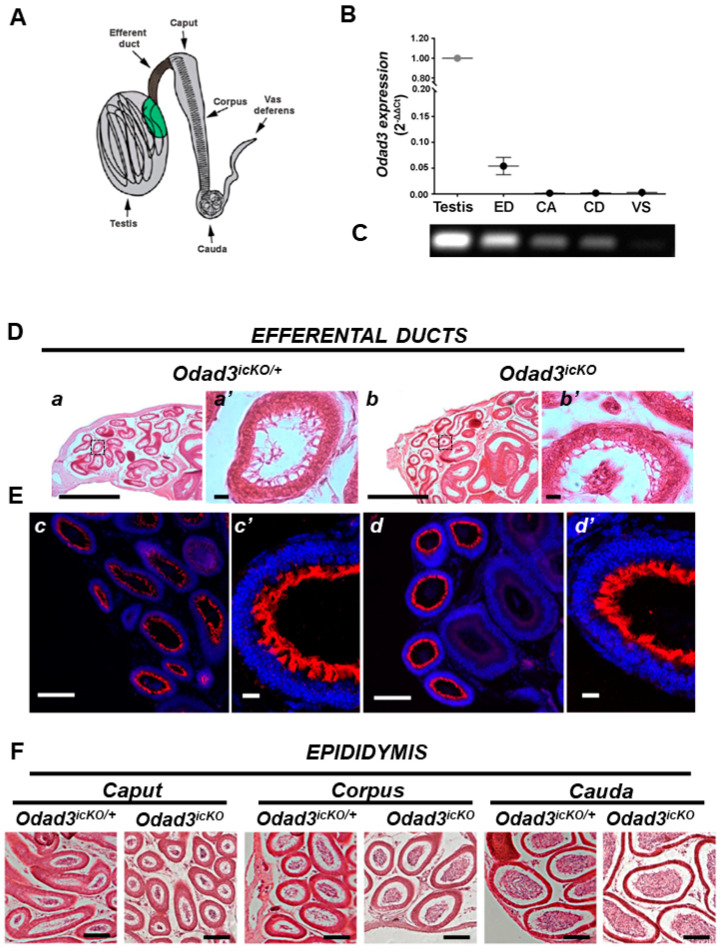
Ablation of the *Odad3* does not affect the ciliogenesis of ED epithelium. (**A**) Schematic representation of the male reproductive system in mice, adopted from Hoque et al., 2021 [19]. The fragments of the reproductive tract dissected for mRNA preparation and q-PCR analysis are indicated by arrows. (**B**) q-PCR analysis of *Odad3* expression in the different regions of the C57BL/6N male reproductive system: mean ± SEM average of the N = 3 independent analysis. ED—efferent ducts; CA—Caput; CD—Cauda; VS—vas deferens. (**C**) *Odad3* q-PCR product separated by gel electrophoresis. One-third of the q-PCR reaction obtained from the testes and an entire q-PCR reaction from the other regions of the male reproductive system were loaded on the gel. (**D**) H&E-stained sections of the efferent ducts: *Odad3^icKO^* (**a**,**a’**) and *Odad3^icKO/+^* (**b**,**b’**). The black boxes are magnified on the right side of the corresponding image. Scale bar: images **a**,**b**—500 μm; images **a’**,**b’**—10 μm. (**E**) IF analysis with anti-acetylated alpha tubulin antibodies of ED cross-sections: *Odad3^icKO^* (**c**,**c’**) and *Odad3^icKO/+^* (**d**,**d’**). The anti-acetylated alpha tubulin staining is red and DAPI-stained nuclei are blue. Scale bar: **c**,**d**—50 μm; **c’**,**d’**—10 μm (**F**) Representative H&E-stained sections from different regions of the reproductive system of *Odad3^icKO^* (N = 3) and *Odad3^icKO/+^* (N = 3) animals. Scale bar: 50 μm.

We investigated the potential impact of *Odad3* gene ablation on ED ciliogenesis. Initially, q-PCR analysis was employed to assess *Odad3* expression within various fragments of reproductive tract in wild-type animals: testis, efferent ducts (ED), caput epididymis (CA), cauda epididymis (CD) and vas deferens (VS) (Figure 5A). This analysis revealed a high expression of the *Odad3* gene in testes, a very low expression in ED, minimal levels in CA and CD and undetectable levels in the VS of the murine reproductive tract (Figure 5B,C). We then performed histological analysis of the ED sections stained with H&E (Figure 5D). The analysis confirmed the presence of cilia on MCCs in both mutant and control animals with no discernible differences in ciliary morphology between the two genotypes. Furthermore, IF staining for acetylated tubulin, a ciliary axoneme marker revealed the presence of the cilia in the ED of animals with the conditional knockout of the *Odad3* gene (Figure 5E). This finding suggests that *Odad3* ablation in adult animals does not perturb the ciliogenesis process in MCCs of the ED. We then histologically analyzed regions of epididymis. No obvious histological differences were observed in the caput, corpus and cauda of mutant and control animals (Figure 5F). 

### 3.6. Heterozygous Odad3 Knockout Animals Are Subfertile

**Figure 6:**
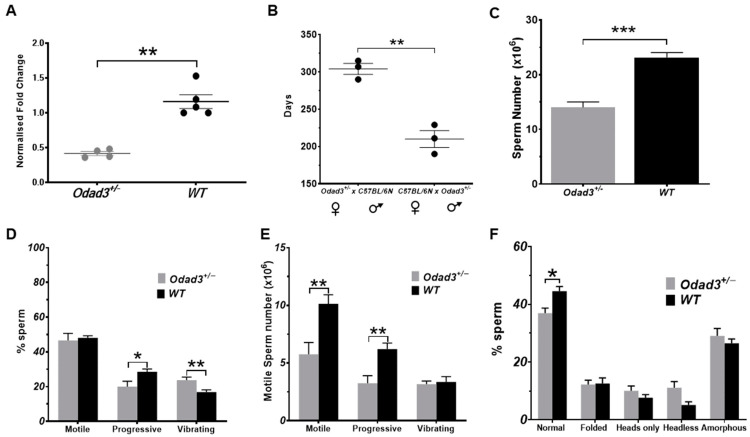
Analysis of fertility in heterozygous *Odad3* knockout males. (**A**) Quantitative PCR analysis of *Odad3* mRNA transcript from adult testes of *Odad3^+/−^* and wild-type (WT) littermates. Mean ± SEM; unpaired two-tailed *t*-test ** *p* = 0.007. (**B**) Fertility testing of the *Odad3^+/−^* animals. The reciprocal breeding of *Odad3^+/−^* females (N = 3) and males (N = 3) with C57BL/6N (background strain) males or females was set up at 8–12 weeks of age. The age (days) at which the last litter laid was plotted. After the last litter was delivered, the couples were maintained together in the breeding cage for at least 3 months. Mean ± SEM; unpaired *t*-test with Welsh’s correction ** *p* = 0.003. (**C**) Sperm count analysis. Spermatozoa from cauda of 8-month-old *Odad3^+/−^* (N = 7) and wild-type (N = 7) littermates were counted; mean ± SEM; unpaired two-tailed *t*-test *** *p* = 0.0002. (**D**) Analysis of the sperm motility. The percentage of the motile, progressively moving, and vibrating sperm prepared from cauda was analyzed; mean ± SEM; N = 7 per genotype/experimental group; unpaired two-tailed *t*-test * *p* = 0.027; ** *p* = 0.007. (**E**) Number of motile spermatozoa prepared from cauda; mean ± SEM; N = 7 per genotype/experimental group; unpaired two-tailed *t*-test; Total motile sperm count ** *p* = 0.005; Total progressive motile sperm count ** *p* = 0.004. (**F**) Morphological analysis of the spermatozoa spreads isolated from cauda. Error bars represent the mean ± SEM; N = 7 per genotype/experimental group; unpaired two-tailed *t*-test * *p* = 0.013. When analyzing *Odad3^+/−^* sperm, we observed trends to increase in the percentage of headless and amorphous sperm. However, the trends are not statistically significant. The category of headless sperm did not reach statistical significance with a *p* value of 0.058.

Our q-PCR data demonstrated that the conditional deletion of the *Odad3* gene was incomplete (Figure 2A). Yet, we have also demonstrated that the quantitative differences in the *Odad3* transcript between *Odad3^icKO^* and *Odad3^icKO/+^* animals lead to phenotypical differences in sperm morphology and motility most likely due to defective spermiogenesis. We, therefore, asked whether the germline transmitted *Odad3* haplo-insufficiency also has a phenotypic manifestation. To do this, we analyzed the fertility parameters in heterozygous *Odad3* knockout males (*Odad3^+/−^*) and wild-type littermates. Firstly, we examined whether the expression of the *Odad3* gene is gene dosage-dependent. To do this, we performed q-PCR analysis of the *Odad3* mRNA isolated from the adult (8-month-old) testes of *Odad3^+/−^* and wild-type littermates. Quantitative analysis revealed a reduction in *Odad3* transcript of approximately 50% in *Odad3^+/−^* testes (Figure 6A). 

Then, we performed a fertility assay using the reciprocal breeding of *Odad3^+/−^* males and females with the C57BL/6N inbred strain. The test revealed a reduced reproductive lifespan in *Odad3^+/−^* males, which ceased to produce the offsprings after 6 months of age. In contrast, *Odad3*^+/−^ females, coupled with C57BL/6N males, maintained reproductive capabilities up to approximately 12 months (Figure 6B), consistent with data from the European Mouse Mutant Archive (EMMA) facility for the C57BL/6N background strain (unpublished data). Our study showed that *Odad3^+/−^* males have a shorter fertile lifespan than *Odad3^+/−^* females and C57BL/6N males. The shorter breeding interval of *Odad3^+/−^* males, also explains the difficulties in maintaining this mutant line. In our hands, the breeding couples ceased to produce the litters in a shorter interval of time, and we periodically had to perform rederivation of the knockout *Odad3* mouse line. This suggests that rapid fertility aging might be caused by *Odad3* gene dosage. 

The breeding test demonstrated that subfertility is caused by a defect present in the male reproductive system. To define the defect, we assessed testicular weight and analyzed mature sperm from the cauda epididymis of 8-month-old males, when fertility decline became evident. No significant difference in testes weights between wild-type and *Odad3^+/−^* littermates was observed (Supplementary Figure S3). However, a significant reduction in total sperm count was revealed in *Odad3^+/−^* males (Figure 6C). The analysis of sperm motility demonstrated that while the percentage of sperm able to move is not affected, a significant decrease in the percentage of sperm with progressive movement and an increase in the percentage of vibrating sperm were observed (Figure 6D). Furthermore, the total number of motile progressively moving sperm was significantly decreased in *Odad3^+/−^* males when compared to wild-type littermates (Figure 6E). The morphological analysis of the sperm revealed a statistically significant decrease in the percentage of sperm with normal morphology in *Odad3^+/−^* males (Figure 6F). Overall, our results show that the decrease in gene dosage leads to oligoastenoteratozoospermia and premature decline of male fertility in *Odad3^+/−^* animals.

## 4. Discussion

Male infertility is a complex condition that affects approximately 7% of worldwide men population. However, the underlying causes are still unknown for almost 40% [45]. In patients with PCD, a genetic mutation in axonemal protein can lead to various types of male infertility, including azoospermia (absence of sperm), oligozoospermia, asthenozoospermia, teratozoospermia, or a combination of the above asthenoteratozoospermia, and oligoasthenoteratozoospermia [46]. It is noteworthy that not all PCD patients present with infertility issues, and PCD infertility is currently under investigation [47]. 

The ODAD3 protein is found in the flagella of sea urchins, bovine, mice and human, suggesting that it is an evolutionarily conserved component of the flagella [20,27,48]. Here, we demonstrated that even partial ablation of the *Odad3* gene in adult murine males might directly lead to MMAF sperm defect. 

In this work, using q-PCR analysis, we demonstrated that the expression of the *Odad3* gene in murine testes during the first wave of spermatogenesis is first detected as early as PND 10 coinciding with the appearance of the first spermatocytes. *Odad3* expression then builds up and is much higher at the time point when the first wave of spermatogenesis produces mature spermatozoa at PND 30–35 and finally reaches its maximum in adult testes. Our findings are consistent with previously published research on the transcriptional profile of murine testes during the first wave of spermatogenesis using next-generation sequencing. The analysis showed that *Odad3* expression is first detectable between postnatal days 7 to 10, coinciding with the appearance of spermatogonia and primary spermatocytes, and reaches its peak at postnatal day 28, corresponding to the elongated spermatids stage [3,49]. The PND 28 stage was the last temporal point investigated in these studies [49]. The presence of ODAD3 protein is also detected in adult human testes [27,50]. Overall, these data allow us to suggest an important role of the *Odad3* gene both in the first wave and in adult spermatogenesis.

The partial ablation of the *Odad3* gene in adult testes leads to a block in spermatogenesis at the spermiation stages. The accumulation of the seminiferous tubules at stages VII–VIII might suggest a delay in the release of spermatozoa from the seminiferous epithelium. Future studies are required to determine the primary defect responsible for the block of spermiation. This block can be explained by defects in the normal axonemal assembly of the manchette, nuclear condensation processes and/or defects in axonemal flagella assembly. Given the Odad3 protein is present in flagella of the murine spermatozoa [48], it is reasonable to hypothesize that the primary consequence of *Odad3* ablation could be the delayed or abnormal assembly of flagella in the testes, which would ultimately result in a delay in the release of the mature sperm during spermiation.

In this study, we have shown that the *Odad3* gene is expressed in ED, although its expression level is notably lower than in testes. We estimated, using q-PCR analysis, that only approximately 5% of the *Odad3* mRNA detected in testes is present in ED. We used histological and IF analysis with anti-acetylated tubulin antibodies and demonstrated that ciliogenesis in ED is not grossly affected following *Odad3* gene ablation. However, we cannot rule out the possibility that the ciliary structure, directional and/or beating frequency of ED cilia may be defective because of the *Odad3* deletion. Such studies conducted on *Dnah5* (axonemal protein) mutant mice have shown that, while cilia were present on MCCs cells of EDs, an abnormal cilia beat frequency leads to asthenozoospermia due to sperm accumulation and agglutination in the ED of *Dnah5* mutant mice. Importantly, the oligozoospermia, due to ED cilia dysmotility, was also observed in patients with *DNAH5* mutations [18].

It has been demonstrated that in mouse models with abnormal ciliogenesis of MCCs in ED, such as those with (1) tissue-specific FoxJ1-Cre knockout of CEP164, a centrosomal protein with an essential role in ciliogenesis and (2) knockout of *miR-34b/c* and *-449a/b/c* clusters, MCCs have no cilia or solo rudimental cilia. In these animals, the EDs were obstructed by agglutinated sperm, and the seminiferous tubules exhibited enlarged lumens and reduced stratification of the seminiferous epithelium, accompanied by a significantly reduced number of spermatozoa [17,19]. In our analysis, we did not observe an enlargement of seminiferous tubules in animals with *Odad3^icKO^*; in contrast, the diameters of the tubules were not changed but showed a significant decrease in free luminal space. After comparing published results and presented analysis of *Odad3^icKO^* testes, we concluded that the possible abnormality of MCCs cilia motility is not the primary cause of subfertility in *Odad3^icKO^* animals. However, we cannot completely rule out the possibility that the complete loss of *Odad3* function, starting from the germline, could block the ciliogenesis of ED MCCs or cause ED cilia abnormal motility, leading to asthenozoospermia, as we observed in surviving mice with a complete knockout of the *Odad3* gene [24]. 

It has recently been demonstrated that the dosage of a specific gene is crucial for normal physiological function and that abnormal levels can lead to various diseases and infertility [51,52,53,54]. In this study, we found that the correct gene dosage of *Odad3* is important for normal spermatogenesis and sperm development using conditional deletion of the *Odad3* gene. Phenotypic sperm analysis was performed in homozygous *Odad3^icKO^* males and heterozygous *Odad3^icKO/+^* animals following the induction of Odad3 gene deletion in adult animals. Our qPCR analysis of *Odad3* expression demonstrated a significant (20–50%) reduction in *Odad3* levels in the testes of *Odad3^icKO^* compared to *Odad3^icKO/+^* animals. The observed phenotypic differences in sperm motility and morphology strongly suggest that *Odad3* gene dosage may play an important role in the processes of sperm development and maturation. While planning the experiments, we did not expect to observe a gene dosage effect. Therefore, we used the heterozygous *Odad3^icKO/+^* animals as a control group. Our studies on heterozygous *Odad3* animals confirmed the importance of the *Odad3* gene dosage. The qPCR analysis demonstrated a 50% decrease in the *Odad3* transcript levels in *Odad3^+/−^* males when compared to wild-type littermates. This indicates that *Odad3^icKO/+^* animals, which we used as controls for *Odad3* gene deletion, are also affected by low gene dosage. However, since we observed a partial deletion of the *Odad3* gene in adult testes, we can conclude that *Odad3^icKO/+^* animals expressed more than 50% of the *Odad3* transcript. Based on this observation, we would like to suggest that the maintenance of the *Odad3* transcript above haploinsufficiency might be an important determinant for normal male fertility. 

Here, we demonstrated that *Odad3* haploinsufficiency causes oligozoospermia with MMAF phenotype and unexpected decline of fertility in aging males. The gene dosage of axonemal genes and those important for flagella or ED cilia motility are rarely studied because the heterozygous animals are often excluded from the analysis. A recent report found that knockout of *Cfap43* and *Cfap44*, two axonemal genes, resulted in severe MMAF phenotypes in mice, while heterozygous *Cfap43^+/−^* mice exhibited impaired progressive sperm motility and *Cfap44^+/−^* mice had a higher proportion of morphologically abnormal spermatozoa [55]. This suggests that the impact of gene dosage of axonemal genes on spermatozoa development and MMAF phenotype requires more attention and investigation. Importantly, it was observed that *Odad3^+/−^* males have a faster fertility decline, suggesting that a combination of a decrease in gene dosage by causing a potential reduction in the *Odad3* protein level and pressure from testicular aging might lead to premature infertility in males with *Odad3* heterozygous loss-of-function mutation. 

Overall, our results suggest that the dosage of axonemal genes might be taken into consideration when investigating the infertility of men with unknown genetic causes. The results also highlight the critical role of the *Odad3* gene in PCD-related male infertility, suggesting the need for genetic counseling of PCD patients with *Odad3* loss-of-function mutations for future practices.

## Data Availability

The original contributions presented in the study are included in the article/supplementary material, further inquiries can be directed to the corresponding authors.

## Supplementary Materials

The following supporting information can be downloaded at: https://www.mdpi.com/article/10.3390/cells13121053/s1, Figure S1: Schematic representation of the knockout *Odad3^tm1b^* (reporter *Odad3-LacZ*) allele (adopted from Chiani et al. [24]). The *Odad3^tm1b^* allele is a knockout of the *Odad3* gene (previously called *Ccdc151* gene) and in the text is labeled as the *Odad3−LacZ* or *Odad3^−^ *allele. *Ex*-exon; *En2A SA*-splice acceptor site; *IRES*-internal ribosomal entry site; *lacZ*-bacterial β-galactosidase reporter gene; *pA*-poly-A; *hBactP*-human β-actin promoter; neo-neomycin resistance gene; *FRT*-FLP recombination sites; *LoxP*-Cre recombination sites. Figure S2: (A) Schematic representation of the conversion of the *Odad3^tm1a^* allele into the conditional allele *Odad3^tm1c^* upon FLP-dependent recombination (adopted from Chini et al. [24]). *Ex*-exon; *En2SA*-splice acceptor site; *IRES*-internal ribosomal entry site; *LacZ*-bacterial β-galactosidase re-porter gene; *pA*-poly-A; *hBactP*-human β-actin promoter; neo-neomycin resistance gene; *FRT*-FLP recombination sites; *LoxP*-Cre recombination sites. (B) The scheme of the ERT2-Cre induction by tamoxifen. The induction was performed by tamoxifen injections delivered to 4-month-old (sexually mature) animals and sperm and organ collection was performed 4 months after tamoxifen injection. This time point has been chosen based on the duration of the spermatogenesis wave in adults. The cycle of spermatogenesis is the time which is required for type A spermatogonia to develop into spermatozoa. In mice, this cycle is 34.5 days [56]. We allowed at least 3 cycles of complete spermatogenesis to ensure that the germ cell with the deletion of the *Odad3* gene entered the spermatogenesis wave and then performed the sperm analysis and histological analysis of the testes. Figure S3: Analysis of testes weight in heterozygous *Odad3^+/−^* males. At the 8-month old males were euthanized and weighed. The testes were dissected and weighted. (A) Testis weight analysis. Experimental animals: *Odad3^+/−^* (N = 7) and wild type (WT) (N = 7). (B). Testis weight was normalized to body weight. Experimental animals: *Odad3^+/−^* (N = 7) and wild type (WT) (N = 7). No significant differences between absolute testis weight and testis/body weight ratio between *Odad3^+/−^* and wild type (WT) animals were observed.

## References

[1] Lyu Q., Li Q., Zhou J., Zhao H. (2024). Formation and function of multiciliated cells. J. Cell Biol..

[2] Wallmeier J., Nielsen K.G., Kuehni C.E., Lucas J.S., Leigh M.W., Zariwala M.A., Omran H. (2020). Motile ciliopathies. Nat. Rev. Dis. Primers.

[3] Sironen A., Shoemark A., Patel M., Loebinger M.R., Mitchison H.M. (2020). Sperm defects in primary ciliary dyskinesia and related causes of male infertility. Cell. Mol. Life Sci..

[4] Fliegauf M., Benzing T., Omran H. (2007). When cilia go bad: Cilia defects and ciliopathies. Nat. Rev. Mol. Cell Biol..

[5] Satir P., Christensen S.T. (2007). Overview of Structure and Function of Mammalian Cilia. Annu. Rev. Physiol..

[6] Klena N., Pigino G. (2022). Structural Biology of Cilia and Intraflagellar Transport. Annu. Rev. Cell Dev. Biol..

[7] Ishikawa T. (2017). Axoneme structure from motile cilia. Cold Spring Harb. Perspect. Biol..

[8] Griswold M.D. (2016). Spermatogenesis: The Commitment to Meiosis. Physiol. Rev..

[9] Oakberg E.F. (1956). A description of spermiogenesis in the mouse and its use in analysis of the cycle of the seminiferous epithelium and germ cell renewal. Am. J. Anat..

[10] Meistrich M.L., Hess R.A., Carrell D.T., Aston K.I. (2013). Assessment of Spermatogenesis through Staging of Seminiferous Tubules. Spermatogenesis.

[11] Hermo L., Pelletier R.-M., Cyr D.G., Smith C.E. (2010). Surfing the wave, cycle, life history, and genes/proteins expressed by testicular germ cells. Part 1: Background to spermatogenesis, spermatogonia, and spermatocytes. Microsc. Res. Tech..

[12] O’Donnell L. (2014). Mechanisms of spermiogenesis and spermiation and how they are disturbed. Spermatogenesis.

[13] Leblond C.P., Clermont Y. (1952). Spermiogenesis of rat, mouse, hamster and guinea pig as revealed by the “periodic acid-fuchsin sulfurous acid” technique. Am. J. Anat..

[14] Hess R. (2015). Small tubules, surprising discoveries: From efferent ductules in the turkey to the discovery that estrogen receptor alpha is essential for fertility in the male. Anim. Reprod..

[15] Hess R.A., Robaire B., Hinton B.T. (2002). The Efferent Ductules: Structure and Functions. The Epididymis: From Molecules to Clinical Practice.

[16] Dacheux J.-L., Dacheux F. (2014). New insights into epididymal function in relation to sperm maturation. Reproduction.

[17] Yuan S., Liu Y., Peng H., Tang C., Hennig G.W., Wang Z., Wang L., Yu T., Klukovich R., Zhang Y. (2019). Motile cilia of the male reproductive system require miR-34/miR-449 for development and function to generate luminal turbulence. Proc. Natl. Acad. Sci. USA.

[18] Aprea I., Nöthe-Menchen T., Dougherty G.W., Raidt J., Loges N.T., Kaiser T., Wallmeier J., Olbrich H., Strünker T., Kliesch S. (2021). Motility of efferent duct cilia aids passage of sperm cells through the male reproductive system. Mol. Hum. Reprod..

[19] Hoque M., Chen D., Hess R.A., Li F.-Q., Takemaru K.-I. (2021). CEP164 is essential for efferent duct multiciliogenesis and male fertility. Reproduction.

[20] Gui M., Farley H., Anujan P., Anderson J.R., Maxwell D.W., Whitchurch J.B., Botsch J.J., Qiu T., Meleppattu S., Singh S.K. (2021). De novo identification of mammalian ciliary motility proteins using cryo-EM. Cell.

[21] Hjeij R., Onoufriadis A., Watson C.M., Slagle C.E., Klena N.T., Dougherty G.W., Kurkowiak M., Loges N.T., Diggle C.P., Morante N.F. (2014). CCDC151 mutations cause primary ciliary dyskinesia by disruption of the outer dynein arm docking complex formation. Am. J. Hum. Genet..

[22] Leung M.R., Zeng J., Wang X., Roelofs M.C., Huang W., Chiozzi R.Z., Hevler J.F., Heck A.J., Dutcher S.K., Brown A. (2023). Structural specializations of the sperm tail. Cell.

[23] Alsaadi M.M., Erzurumluoglu A.M., Rodriguez S., Guthrie P.A.I., Gaunt T.R., Omar H.Z., Mubarak M., Alharbi K.K., Al-Rikabi A.C., Day I.N.M. (2014). Nonsense Mutation in Coiled-Coil Domain Containing 151 Gene (*CCDC151*) Causes Primary Ciliary Dyskinesia. Hum. Mutat..

[24] Chiani F., Orsini T., Gambadoro A., Pasquini M., Putti S., Cirilli M., Ermakova O., Tocchini-Valentini G.P. (2019). Functional loss of Ccdc151 leads to hydrocephalus in a mouse model of primary ciliary dyskinesia. Dis. Models Mech..

[25] Demir Eksi D., Yilmaz E., Basaran A.E., Erduran G., Nur B., Mihci E., Karadag B., Bingol A., Alper O.M. (2022). Novel Gene Variants Associated with Primary Ciliary Dyskinesia. Indian J. Pediatr..

[26] Zhang W., Li D., Wei S., Guo T., Wang J., Luo H., Yang Y., Tan Z. (2019). Whole-exome sequencing identifies a novel CCDC151 mutation, c. 325G> T (p. E109X), in a patient with primary ciliary dyskinesia and situs inversus. J. Hum. Genet..

[27] Greither T., Dejung M., Behre H.M., Butter F., Herlyn H. (2023). The human sperm proteome—Toward a panel for male fertility testing. Andrology.

[28] Kos R., Israëls J., Van Gogh C.D.L., Altenburg J., Diepenhorst S., Paff T., Boon E.M.J., Micha D., Pals G., Neerincx A.H. (2022). Primary ciliary dyskinesia in Volendam: Diagnostic and phenotypic features in patients with a *CCDC114* mutation. Am. J. Med. Genet. Part C Semin. Med. Genet..

[29] Onoufriadis A., Paff T., Antony D., Shoemark A., Micha D., Kuyt B., Schmidts M., Petridi S., Dankert-Roelse J.E., Haarman E.G. (2013). Splice-site mutations in the axonemal outer dynein arm docking complex gene CCDC114 cause primary ciliary dyskinesia. Am. J. Hum. Genet..

[30] Gao Y., Xu C., Tan Q., Shen Q., Wu H., Lv M., Li K., Tang D., Song B., Xu Y. (2021). Case report: Novel biallelic mutations in ARMC4 cause primary ciliary dyskinesia and male infertility in a Chinese family. Front. Genet..

[31] Hjeij R., Lindstrand A., Francis R., Zariwala M.A., Liu X., Li Y., Damerla R., Dougherty G.W., Abouhamed M., Olbrich H. (2013). ARMC4 mutations cause primary ciliary dyskinesia with randomization of left/right body asymmetry. Am. J. Hum. Genet..

[32] Onoufriadis A., Shoemark A., Munye M.M., James C.T., Schmidts M., Patel M., Rosser E.M., Bacchelli C., Beales P.L., Scambler P.J. (2013). Combined exome and whole-genome sequencing identifies mutations in ARMC4 as a cause of primary ciliary dyskinesia with defects in the outer dynein arm. J. Med. Genet..

[33] Wang K., Chen X., Guo C.Y., Liu F.Q., Wang J.R., Sun L.F. (2018). Cilia ultrastructural and gene variation of primary ciliary dyskinesia: Report of three cases and literatures review. Zhonghua Er Ke Za Zhi=Chin. J. Pediatr..

[34] Cheng W., Ip Y.T., Xu Z. (2013). Gudu, an Armadillo repeat-containing protein, is required for spermatogenesis in Drosophila. Gene.

[35] Backman K., Mears W.E., Waheeb A., Bergeron M.B., McClintock J., De Nanassy J., Reisman J., Osmond M., Hartley T., Mears A.J. (2021). A splice site and copy number variant responsible for TTC25-related primary ciliary dyskinesia. Eur. J. Med. Genet..

[36] Hjeij R., Aprea I., Olbrich H., Dougherty G.W., Omran H. (2021). Defects in Outer Dynein Arm Docking Machinery Cause Primary Ciliary Dyskinesia.

[37] Wallmeier J., Shiratori H., Dougherty G.W., Edelbusch C., Hjeij R., Loges N.T., Menchen T., Olbrich H., Pennekamp P., Raidt J. (2016). TTC25 deficiency results in defects of the outer dynein arm docking machinery and primary ciliary dyskinesia with left-right body asymmetry randomization. Am. J. Hum. Genet..

[38] He H., Yu F., Shen W., Chen K., Zhang L., Lou S., Zhang Q., Chen S., Yuan X., Jia X. (2021). The novel key genes of non-obstructive azoospermia affect spermatogenesis: Transcriptomic analysis based on RNA-Seq and scRNA-Seq Data. Front. Genet..

[39] Ermakova O., Orsini T., Fruscoloni P., Chiani F., Gambadoro A., Putti S., Cirilli M., Mezzi A., Kaciulis S., Pasquini M. (2021). Three-dimensional X-ray imaging of β-galactosidase reporter activity by micro-CT: Implication for quantitative analysis of gene expression. Brain Sci..

[40] Elia J., Imbrogno N., Delfino M., Mazzilli R., Rossi T., Mazzilli F. (2010). The importance of the sperm motility classes-future directions. Open Androl. J..

[41] Oatley J.M., Griswold M.D. (2017). The Biology of Mammalian Spermatogonia.

[42] Hamer G., Roepers-Gajadien H.L., Van Duyn-Goedhart A., Gademan I.S., Kal H.B., Van Buul P.P.W., De Rooij D.G. (2003). DNA Double-Strand Breaks and γ-H2AX Signaling in the Testis1. Biol. Reprod..

[43] Guidi L.G., Holloway Z.G., Arnoult C., Ray P.F., Monaco A.P., Molnár Z., Velayos-Baeza A. (2018). AU040320 deficiency leads to disruption of acrosome biogenesis and infertility in homozygous mutant mice. Sci. Rep..

[44] Nakata H., Wakayama T., Takai Y., Iseki S. (2015). Quantitative Analysis of the Cellular Composition in Seminiferous Tubules in Normal and Genetically Modified Infertile Mice. J. Histochem. Cytochem..

[45] Krausz C., Riera-Escamilla A. (2018). Genetics of male infertility. Nat. Rev. Urol..

[46] Shahrokhi S.Z., Salehi P., Alyasin A., Taghiyar S., Deemeh M.R. (2020). Asthenozoospermia: Cellular and molecular contributing factors and treatment strategies. Andrologia.

[47] Newman L., Chopra J., Dossett C., Shepherd E., Bercusson A., Carroll M., Walker W., Lucas J.S., Cheong Y. (2023). The impact of primary ciliary dyskinesia on female and male fertility: A narrative review. Hum. Reprod. Update.

[48] Zhou L., Liu H., Liu S., Yang X., Dong Y., Pan Y., Xiao Z., Zheng B., Sun Y., Huang P. (2023). Structures of sperm flagellar doublet microtubules expand the genetic spectrum of male infertility. Cell.

[49] Laiho A., Kotaja N., Gyenesei A., Sironen A. (2013). Transcriptome profiling of the murine testis during the first wave of spermatogenesis. PLoS ONE.

[50] Fagerberg L., Hallström B.M., Oksvold P., Kampf C., Djureinovic D., Odeberg J., Habuka M., Tahmasebpoor S., Danielsson A., Edlund K. (2014). Analysis of the human tissue-specific expression by genome-wide integration of transcriptomics and antibody-based proteomics. Mol. Cell. Proteom..

[51] Nivarthi H., Prchal-Murphy M., Swoboda A., Hager M., Schlederer M., Kenner L., Tuckermann J., Sexl V., Moriggl R., Ermakova O. (2015). Stat5 gene dosage in T cells modulates CD8^+^ T-cell homeostasis and attenuates contact hypersensitivity response in mice. Allergy.

[52] Ermakova O., Salimova E., Piszczek L., Gross C. (2012). Construction and phenotypic analysis of mice carrying a duplication of the major histocompatibility class I (MHC-I) locus. Mamm. Genome.

[53] Ermakova O., Piszczek L., Luciani L., Cavalli F.M.G., Ferreira T., Farley D., Rizzo S., Paolicelli R.C., Al-Banchaabouchi M., Nerlov C. (2011). Sensitized phenotypic screening identifies gene dosage sensitive region on chromosome 11 that predisposes to disease in mice. EMBO Mol. Med..

[54] Signore F., Gulìa C., Votino R., De Leo V., Zaami S., Putignani L., Gigli S., Santini E., Bertacca L., Porrello A. (2019). The role of number of copies, structure, behavior and copy number variations (CNV) of the Y chromosome in male infertility. Genes.

[55] Coutton C., Vargas A.S., Amiri-Yekta A., Kherraf Z.-E., Ben Mustapha S.F., Le Tanno P., Wambergue-Legrand C., Karaouzène T., Martinez G., Crouzy S. (2018). Mutations in CFAP43 and CFAP44 cause male infertility and flagellum defects in Trypanosoma and human. Nat. Commun..

[56] Oakberg E.F. (1956). Duration of spermatogenesis in the mouse and timing of stages of the cycle of the seminiferous epithelium. Am. J. Anat..

