# Supplementary material for: The Odad3 Gene Is Necessary for Spermatozoa Development and Male Fertility in Mice
Source: Cells. 2024 Jun 18;13(12):1053. doi: 10.3390/cells13121053 (PMC11201558; doi:10.3390/cells13121053)
Supplement: Supplementary file 1 [file cells-13-01053-s001.zip › cells-3007277-supplementary.pdf]

cells

MDPI

The *Odad3* gene is necessary for spermatozoa development and male fertility in mice

Supplementary Materials:

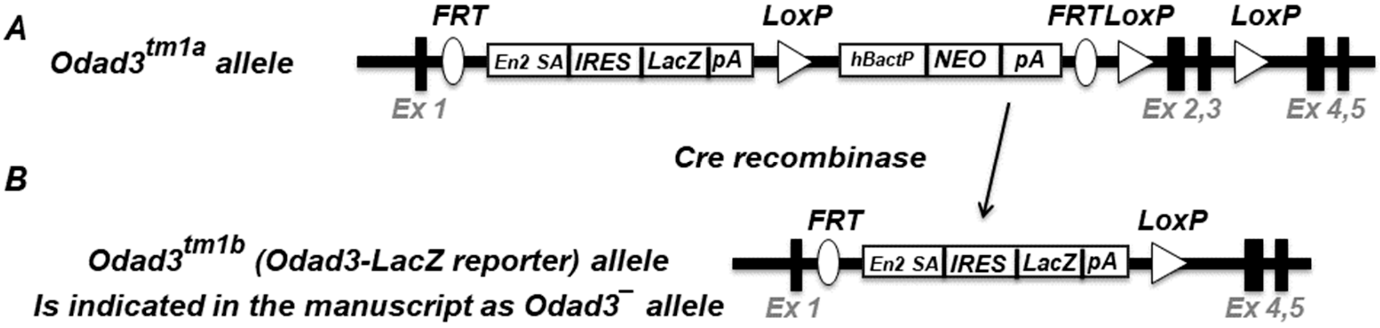

Supplementary Figure S1. Schematic representation of the knockout *Odad3^{tm1b}* (reporter *Odad3-LacZ*) allele (adopted from Chiani et al. [24])**.** The *Odad3^{tm1b}* allele is a knockout of the *Odad3* gene (previously called *Ccdc151* gene) and in the text is labeled as the *Odad3–LacZ* or *Odad3^{-}* allele. *Ex* - exon; *En2A SA* - splice acceptor site; *IRES* - internal ribosomal entry site; *lacZ* - bacterial β-galactosidase reporter gene; *pA* - poly-A; *hBactP* - human β-actin promoter; neo - neomycin resistance gene; *FRT* - FLP recombination sites; *LoxP* - Cre recombination sites.

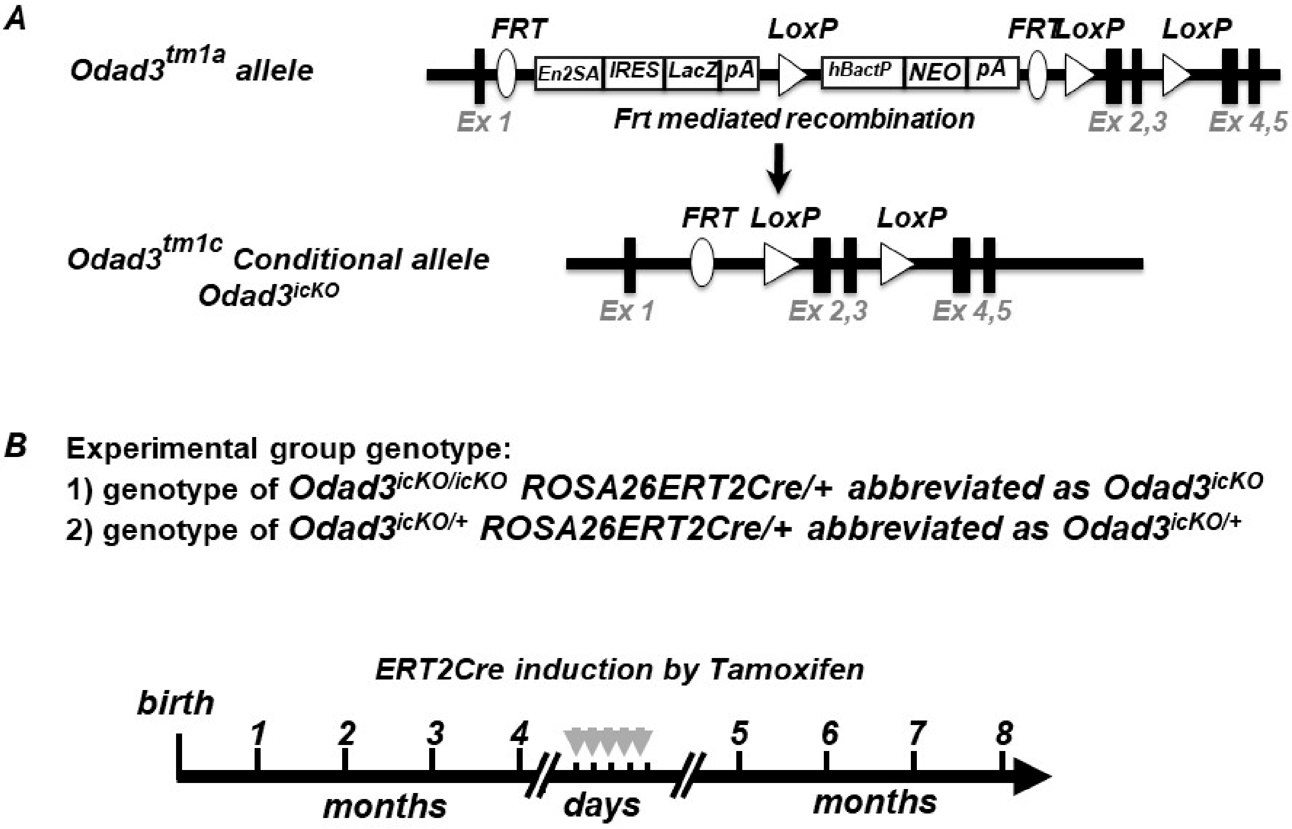

Supplementary Figure S2. (**A**) Schematic representation of the conversion of the *Odad3*^{tm1a} allele into the conditional allele *Odad3*^{tm1c} upon FLP-dependent recombination (adopted from Chini et al. [24]). *Ex* - exon; *En2SA* - splice acceptor site; *IRES* - internal ribosomal entry site; *LacZ* - bacterial β-galactosidase re-porter gene; *pA* - poly-A; *hBactP* - human β-actin promoter; neo - neomycin resistance gene; *FRT* - FLP recombination sites; *LoxP* - Cre recombination sites. (**B**) The scheme of the ERT2-Cre induction by tamoxifen. The induction was performed by tamoxifen injections delivered to 4-month-old (sexually mature) animals and sperm and organ collection was performed 4 months after tamoxifen injection. This time point has been chosen based on the duration of the spermatogenesis wave in adults. The cycle of spermatogenesis is the time which is required for type A spermatogonia to develop into spermatozoa. In mice, this cycle is 34,5 days [56]. We allowed at least 3 cycles of complete spermatogenesis to ensure that the germ cell with the deletion of the *Odad3* gene entered the spermatogenesis wave and then performed the sperm analysis and histological analysis of the testes.

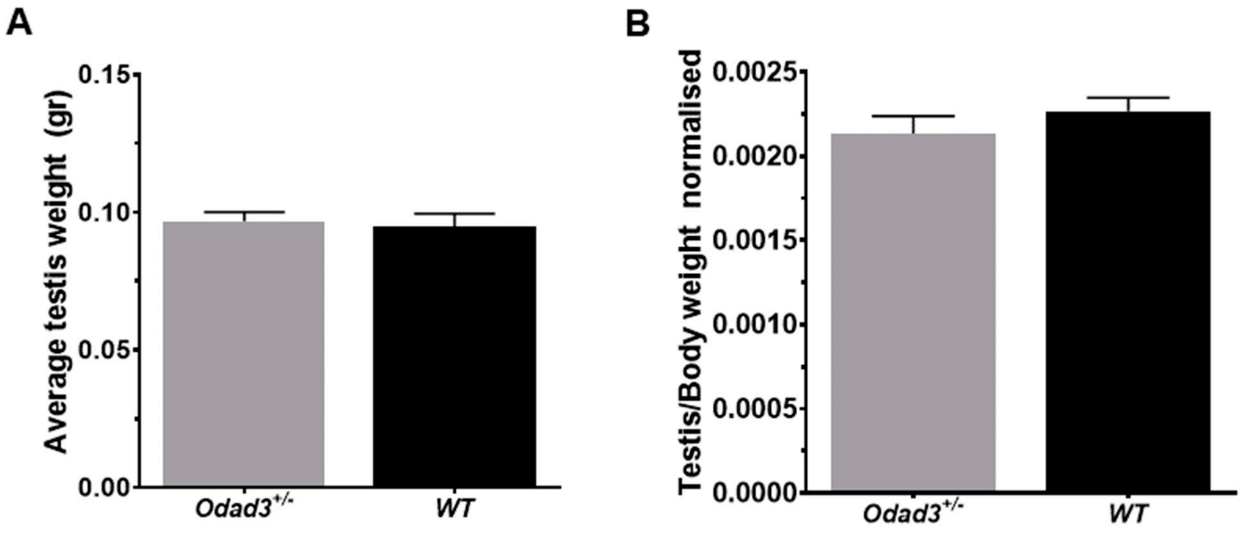

Supplementary Figure S3. Analysis of testes weight in heterozygous *Odad3*$^{+/-}$ males. At the 8-month old males were euthanized and weighed. The testes were dissected and weighed. (**A**) Testes weight analysis. Experimental animals: *Odad3*$^{+/-}$ (N=7) and wild type (WT) (N=7). (**B**). Testis weight was normalized to body weight. Experimental animals: *Odad3*$^{+/-}$ (N=7) and wild type (WT) (N=7). No significant differences between absolute testis weight and testis/body weight ratio between *Odad3*$^{+/-}$ and wild type (WT) animals were observed.
